# Case Report: Rupture of the cardiac free wall after myocardial infarction confirmed by aortic computed tomography angiography

**DOI:** 10.3389/fcvm.2025.1502336

**Published:** 2025-06-19

**Authors:** Jie Liu, Weiqing Chen, Zihan Li, Nan Liu, Cuiying Mao

**Affiliations:** Department of Cardiology, Jilin Provincial Engineering Laboratory for Endothelial Function, China-Japan Union Hospital of Jilin University, Changchun, Jilin, China

**Keywords:** cardiac rupture, free wall rupture, aortic computed tomography angiography, diagnosis, myocardial infarction

## Abstract

**Introduction:**

Free wall rupture is a rare complication of myocardial infarction, occurring as early as a few hours after myocardial infarction. It usually happens after transmural myocardial infarction and has a very high mortality rate. The diagnosis of free wall rupture requires echocardiographic evidence of pericardial leakage or pericardial tamponade. The patient's cause of death is often attributed to other causes such as cardiac arrhythmia unless determined by open heart surgery or autopsy. Diagnostic tools for post-infarction cardiac free wall rupture are limited to date.

**Case presentation:**

We present a case of myocardial infarction in a patient who presented with atypical chest pain. Subsequent coronary angiography revealed small vessel disease, however, the patient's severe clinical presentation was not consistent with small vessel disease. The initial clinical presentation did not rule out aortic dissection, prompting further investigation through aortic computed tomography. This imaging technique revealed a rupture of the free wall of the heart accompanied by a large amount of bloody pericardial effusion. Unfortunately, attempts to puncture and drain the effusion were unsuccessful and the patient eventually succumbed.

**Conclusions:**

When patients present with more severe clinical manifestations that are not consistent with myocardial infarction, it is important to be alert to the possibility of cardiac rupture in addition to identifying the possibility of aortic dissection. Aortic CTA may be able to confirm the diagnosis of cardiac rupture.

## 1 Introduction

Cardiac rupture is one of the complications of myocardial infarction and is often characterized by acute episodes of shortness of breath, chest pain, shock, sweating, unexplained vomiting, cold clammy skin, and fainting (1). Risk factors for cardiac rupture after myocardial infarction include older adults, women, hypertension (2–6), single-vessel disease, and first-time infarction (7, 8). Pericardial effusion is the most common manifestation of cardiac rupture during acute myocardial infarction. It has extremely limited diagnostic tools and can often only be clarified by open-heart surgery or occasionally by echocardiography or angiography showing blood leakage into the pericardial cavity (9). A ruptured heart can lead to rapid death, making prompt diagnosis and treatment crucial1. We will present a patient with myocardial infarction who had a rupture of the free wall of the heart detected not by cardiac ultrasound or coronary angiography but by aortic computed tomography angiography (CTA).

## 2 Case presentation

**Figure 1:**
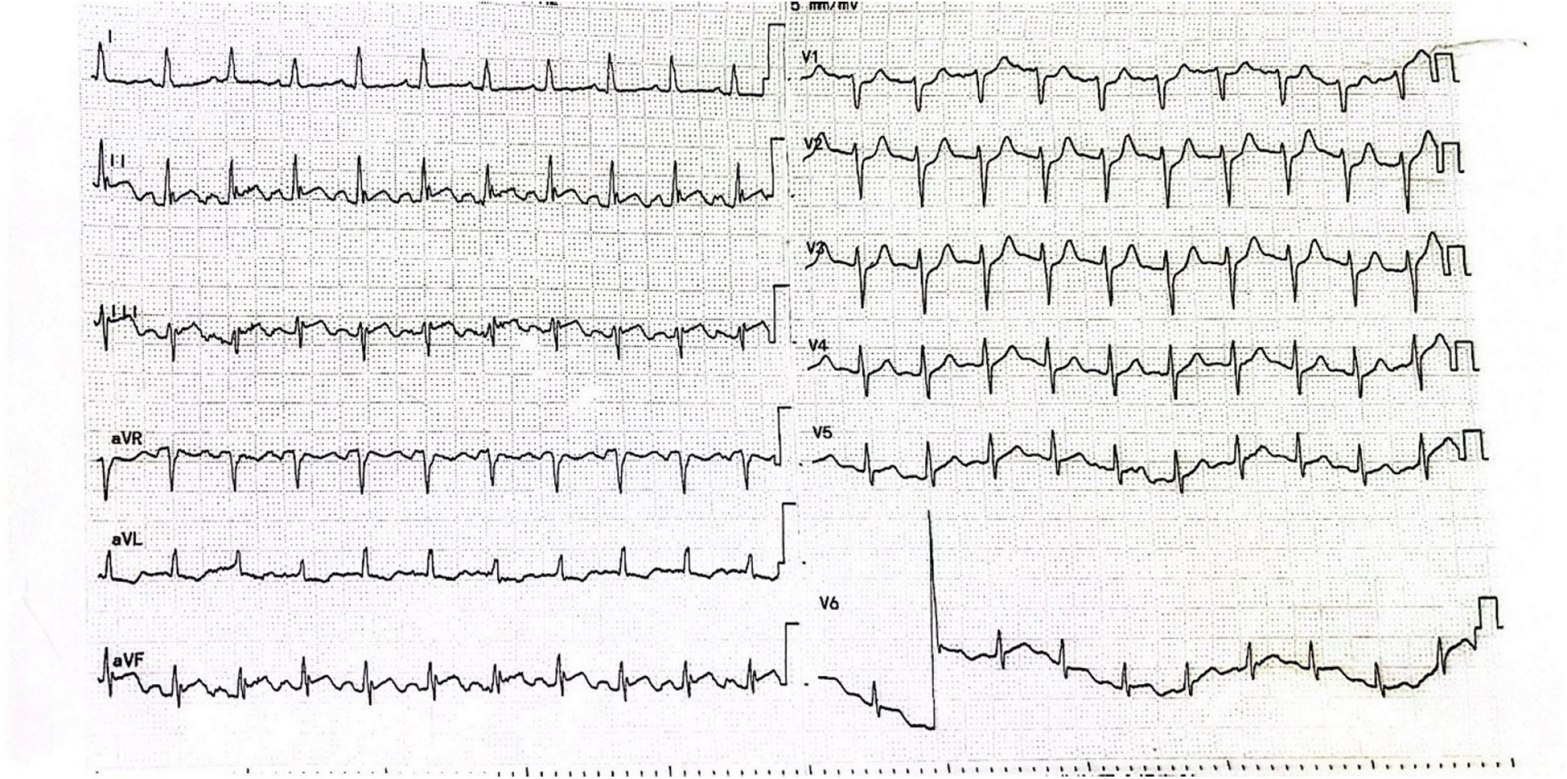
Patient's electrocardiogram on admission.

We report a 47-year-old middle-aged male admitted to the emergency department with severe posterior back and lumbar pain. The patient's general state was poor, characterized by agitation, dyspnea, inability to lie down, cyanosis, and clammy cold skin. Electrocardiogram showed ST-segment elevation in leads II, III and AVF (Figure 1). Based on the patient's symptoms and history of hypertension it was considered that aortic dissection was not excluded and that the elevation of the ST segment in the inferior wall lead could be due to a cumulative right coronary artery opening. It was then decided to further identify the patient by drawing blood for laboratory tests.

**Figure 2:**
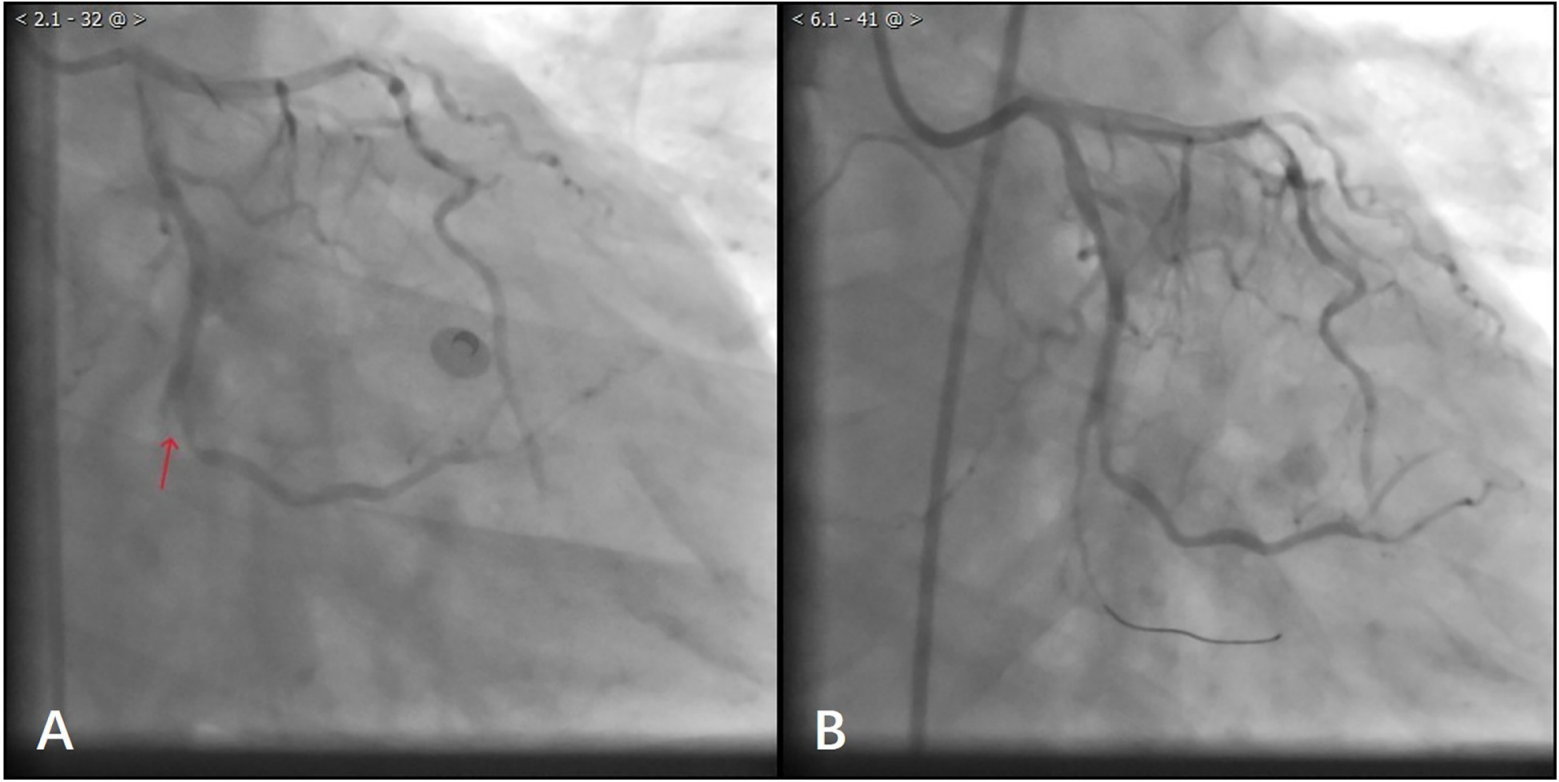
**(A)** Patient with angiographic occlusion of vessels distal to the gyratory branch. **(B)** Blood flow through the distal vessels of the gyratory branch after balloon dilatation.

After waiting for 10 min, the laboratory results showed TroponinI 0.87 ng/ml (0.01–0.023), D-dimer: 978 ng/ml (80–500), N-Terminal Pro-Brain Natriuretic Peptide 599 ng/L (300–500), Lactate 25 mmol/L (0.5–1.6), Glucose 30 mmol/L (3.9–5.8), Potassium 2.7 mmol/L (3.4–4.5). Considering that the patient's troponin was markedly elevated and his D-dimer was not, the decision was made to proceed with coronary arteriography. The coronary angiography of the patient showed normal left coronary artery opening and main body; more than 70% systolic stenosis in the middle muscular bridge of the left anterior descending branch, about 50% stenosis in the middle of the diagonal branch, TIMI flow grade 3; normal opening of the left coronary artery circumflex, no obvious stenosis in the main body, distal occlusion, TIMI flow grade 0; normal opening of the right coronary artery, 30%–40% stenosis in the middle, TIMI flow grade 3 (Figure 2A). During the operation, the patient's blood pressure decreased, and we gave fluid replacement and continuous noninvasive ventilator-assisted breathing. The vessels in the distal segment of the occluded circumflex were small (Figure 2A), which was inconsistent with the clinical manifestations of critical illness. Considering the possibility of aortic dissection, an urgent aortic CTA was performed.

**Figure 3:**
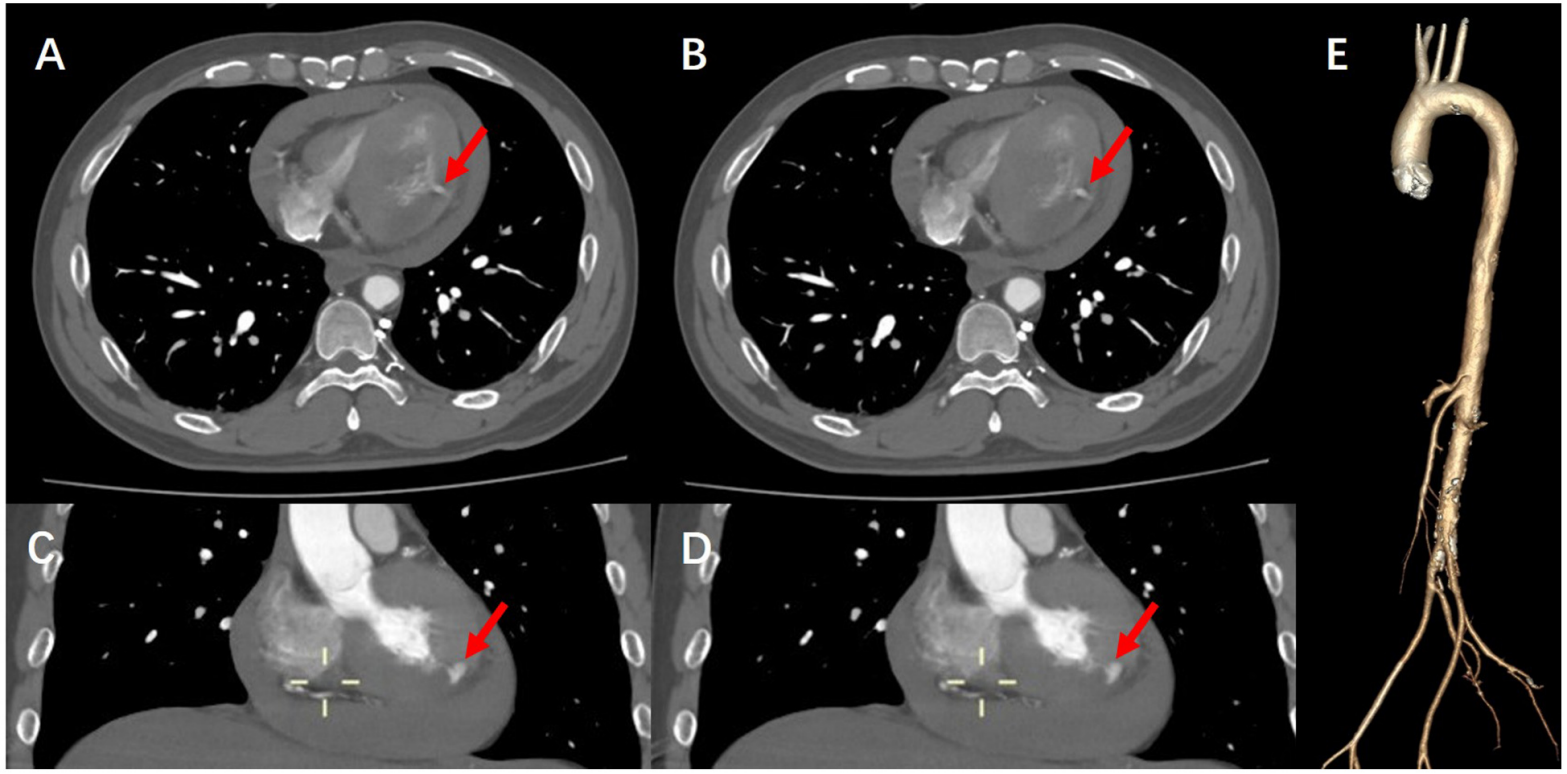
**(A,B)** CTA of the aorta demonstrate contrast extravasation into the myocardium, along with right-sided heart failure and pericardial effusion. **(C,D)** Coronal views of aortic CTA further reveal contrast leakage into the myocardial tissue. **(E)** Three-dimensional reconstruction of the aorta confirms the absence of aortic dissection, intramural hematoma, or other vascular anomalies.

During this period, although the patient underwent echocardiography, the bedside ultrasound did not find pericardial tamponade, but only moderate amount of pericardial effusion due to the patient's obesity, restlessness and the limitations of the ultrasound operator's level. Subsequent aortic CTA revealed a significant volume of pericardial effusion without evidence of aortic dissection. The clinical manifestations were initially attributed to the combined effects of pericardial effusion and coronary pathology. To further clarify the cause of death, a senior radiologist meticulously reviewed the imaging studies and identified contrast medium extravasation from the myocardium into the pericardial cavity (Figure 3). After aortic dissection was ruled out, the patient was returned to the cath lab for further treatment of distal circumflex branch occlusion and pericardiocentesis. Procedure: The guide wire was sent through the catheter and passed through the distal end of the circumflex branch occlusion segment successfully. Blood flow passed through the circumflex branch occlusion segment after balloon expansion. The TIMI grade was 3 (Figure 2B). Unfortunately, pericardiocentesis under DSA guidance was not successful.

**Figure 4:**
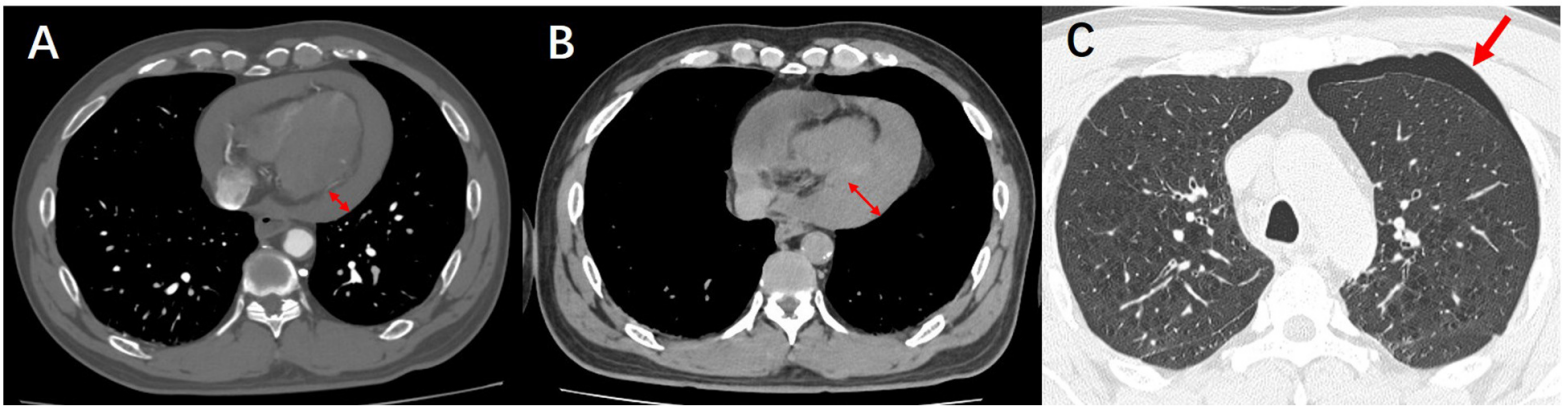
**(A)** CTA demonstrates pericardial effusion. **(B)** Follow-up lung CT reveals an increased volume of pericardial effusion. **(C)** Follow-up lung CT shows newly developed pneumothorax.

The patient's symptoms of generalized cyanosis and dyspnea have been increasing. The patient was then transferred to the cardiac care unit (CCU) for ultrasound-guided pericardiocentesis. After transfer to the intensive care unit, the patient's ultrasound-guided pericardiocentesis remained unsuccessful due to a large amount of gas interference in the chest cavity. Given the possibility of pneumothorax, the patient underwent a computed tomography (CT) scan of the lungs (Figure 4). Pulmonary CT indicated pneumothorax, increased pericardial effusion, and more collapsed right ventricle. Upon transfer back to the intensive care unit, the patient suddenly experienced respiratory arrest, coma, slowed heart rate, and undetectable blood pressure. Emergency rescue of the patient, unfortunately, the patient did not rescue successfully, and eventually died.

## 3 Discussion

Cardiac rupture is one of the most serious complications of acute myocardial infarction. Although its incidence is only 2%–4%, the mortality rate is as high as 90%. This higher mortality rate is closely related to pericardial tamponade (9). Cardiac rupture is not a sudden full-layer rupture of the myocardium. Instead, a rupture occurs in the endocardium first, and blood flows from the rupture into the myocardium to form a myocardial entrapment hematoma, which gradually penetrates to the epicardium, and ultimately ruptures the whole layer to occur pericardial pressure occlusion.

Once a heart rupture has occurred, timely and accurate diagnosis is important (1). Patients presenting with acute catastrophic rupture usually die immediately and are often diagnosed by autopsy (10). In some patients, the clinical manifestation of the subacute process is accompanied by persistent or recurrent severe chest pain after acute myocardial infarction, with entrapment hematoma in the myocardium, and a drop in blood pressure and deterioration of the condition. The blood pressure continues to remain at a low level for several hours to more than ten hours, with a gradual accumulation of seeping blood in the pericardial cavity, followed by the phenomenon of pericardial tamponade.

Early diagnosis and timely emergency surgical treatment of such patients can be expected to be successful. Given its convenience and rapid availability, echocardiography remains recognized as the first-line diagnostic modality for cardiac rupture (11, 12). Although echocardiography demonstrates moderate sensitivity (70%) and high specificity (90%) in detecting cardiac rupture, its diagnostic accuracy heavily depends on operator expertise and patient cooperation (13), which can be particularly challenging in critically ill patients. Therefore, when echocardiography fails to provide a definitive diagnosis, supplementary imaging modalities should be considered. In our case, no extravasation of contrast media was found in the patient during coronary angiography, but the clinical manifestations were inconsistent with coronary vascular disease. However, in order to rule out aortic dissection, we performed an aortic CTA examination and observed contrast agent shuttling through the myocardium and reaching the pericardium, definitively diagnosing the patient's heart rupture diagnosis.

Unfortunately, at the initial diagnosis, we did not alert the patient to the possibility of heart rupture and did not perform pericardial puncture in time. When DSA and cardiac ultrasound were not successfully drained, we considered surgery for the patient, but unfortunately the available surgeon did not arrive at the hospital and the patient died.

This case suggests that when coronary angiography and echocardiography fail to detect heart rupture, especially when there are high risk factors for heart rupture, we may be able to detect heart rupture in patients with myocardial infarction through aortic CTA. At the same time, we should be alert to the possibility of heart rupture when the angiography results are small coronary artery lesions, but the patient has critical clinical manifestations.

## Data Availability

The original contributions presented in the study are included in the article/Supplementary Material, further inquiries can be directed to the corresponding author.
